# Assessing coverage of interventions for reproductive, maternal, newborn, child, and adolescent health and nutrition

**DOI:** 10.1136/bmj.l6915

**Published:** 2020-01-26

**Authors:** Jennifer Requejo, Theresa Diaz, Lois Park, Doris Chou, Allysha Choudhury, Regina Guthold, Debra Jackson, Ann-Beth Moller, Jean-Pierre Monet, Allisyn C Moran, Lale Say, Kathleen L Strong, Anshu Banerjee

**Affiliations:** 1 Unicef, New York, USA; 2 World Health Organization, Geneva, Switzerland; 3 Johns Hopkins University, Baltimore, USA; 4 University of Southern California, Los Angeles, USA; 5 University of Western Cape, Cape Town, South Africa; 6 UNFPA, New York, USA; 7 United Nations H6+ Technical Group, New York, USA,; Competing interests: We have read and understood BMJ policy on declaration of interests and have no relevant interests to declare. The authors alone are responsible for the views expressed in this article, which does not necessarily represent the views, decisions, or policies of the institutions with which the authors are affiliated. Funding from USAID and DFID was used to help support some analysis, graphic design, and to organise references.

## Abstract

Progress has been made in priority interventions, but we need new measurement systems that include the whole life course and give better assessment of equity of coverage, argue **Jennifer Requejo and colleagues**

Embedded within the framework of the United Nations sustainable development goals (SDG) for 2030 is the principle of equity, with the aim of reaching universal health coverage. Soon after the framework was adopted in 2015, the Every Woman Every Child global strategy for women’s, children’s and adolescents’ health was launched.^1^ ^2^ The global strategy translates the SDG agenda into concrete guidance on how to accelerate progress in women’s, children’s and adolescents’ health through a multisectoral approach. It includes a monitoring framework with 60 indicators to help countries and their partners promote accountability in ending preventable deaths (survive), ensuring health and wellbeing (thrive), and expanding enabling environments, so that all women, children, and adolescents can reach their potential (transform).^3^ Previous assessments show mixed progress, with some indicators advancing more rapidly than others but with pervasive inequities between and within countries.^4^

The Countdown to 2030 initiative also regularly tracks progress in the countries experiencing the highest burdens of maternal and child mortality. Countdown’s 2017 report,^5^ together with those from Every Woman Every Child, highlight laudable reductions in maternal and child mortality over the past two decades, but many settings will need to increase efforts to achieve the 2030 goals. Coverage of essential health interventions is unacceptably low in many contexts and among specific populations; considerable policy and programmatic work is needed to shore up primary healthcare systems to make universal health coverage a reality.^5^

We combine the countdown and global strategy indicators to take stock of progress in all 138 low and middle income countries plus Panama, which was reclassified as a high income country in 2018 but remains a priority country in Countdown to 2030.^6^ We examine how well the world is doing in reaching every woman, child, and adolescent with effective health interventions, how far we need to go to achieve the SDGs, and identify gaps in the data. We also propose revisions to the Countdown chart of key indicators so that it better reflects the dimensions of survive, thrive, and transform in the Every Woman Every Child strategy and the interconnecting links between reproductive, maternal, newborn, child, and adolescent health and nutrition. 

Our assessment is based on the common evaluation framework underlying the countdown and global strategy analyses (see web supplement). This framework posits that health outcomes are determined by the ability of healthcare systems to deliver high quality services to all, which, in turn, depends on supportive policies and sufficient resources, including financial, supplies and equipment, and healthcare workers. The framework also considers contextual factors, such as humanitarian and environmental crises, women’s social status, and other political and economic factors that influence access to services, and the independent effects of education and other life opportunities on health.

## Understanding gaps and success

Multiple analytical lenses are needed to understand whether women, children, and adolescents worldwide are receiving effective health interventions. Snapshots of current status tell us where we are and how far we need to go to reach global goals. However, it’s important to visualise trends to determine whether progress is accelerating, stagnating, or even reversing. Countdown’s indicator list helps determine which specific interventions are reaching women, children, and adolescents better than others. Another approach involves ranking countries and regions to identify those that are performing best and those that are lagging behind, providing insight into which countries and regions need constructive action to direct resources and support.

## Where are we now?

**Fig 1:**
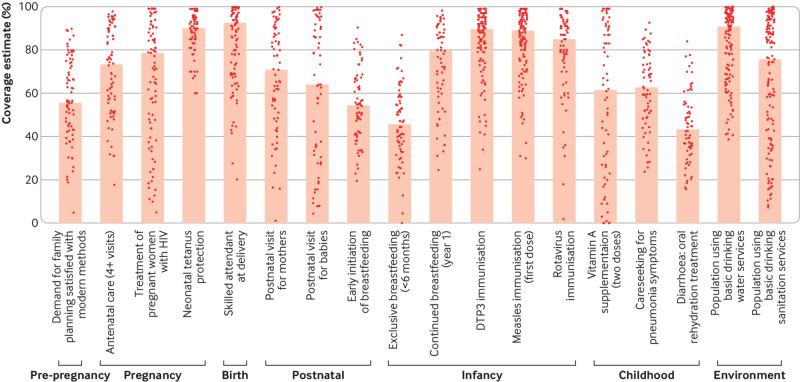
Median national coverage for selected interventions for women’s and child health using most recent WHO and Unicef data for each country (2014 or later; see web supplement for further details).^7^ ^8^ Bars represent the global median and the dots represent country estimates (DTP3=diphtheria-tetanus-pertussis)

Countdown’s continuum of care chart shows a subset of prioritised indicators of intervention coverage across the dimensions of family planning, pregnancy, childbirth, postnatal care, and childhood, with selected crosscutting indicators for water and sanitation. These indicators reflect population level data, showing how well countries are doing in reaching those in need. Figure 1 shows progress on this chart for all low and middle income countries for 2014-18.^7^ ^8^ Summary data (table 1) show that we are far from achieving universal coverage for many interventions, with larger gaps for family planning services, breastfeeding, and treatment of childhood illnesses. Over 80% coverage has been achieved for long established immunisation interventions, skilled attendant at delivery, and safe drinking water, yet coverage is below 50% for interventions that require substantial behaviour change (eg, breastfeeding) or have received less consistent political commitment and resources (eg, oral rehydration salts for diarrhoea).

**Table 1:** Coverage of priority indicators for women’s and children’s health in low and middle income countries* 2014-18^7^

Indicator	No of countries with data	Coverage			Countries with lowest value	Countries with highest value
		Median (interquartile range) coverage (%)	Minimum (%)	Maximum (%)		
Pre-pregnancy						
Demand for family planning satisfied with modern methods	77	56 (42-74)	5	90	Albania	North Korea
Pregnancy						
Antenatal care (4+ visits)	74	73 (53-9)	18	98	Afghanistan	Cuba
Treatment of pregnant women living with HIV	93	79 (46-93)	5	99	Sudan	Benin, Bolivia, Cuba, Grenada, Jamaica, Mozambique, Mauritius, Malawi, Malaysia, Namibia, Romania, Rwanda, Sao Tome and Principe, Suriname, Saint Vincent and the Grenadines, Zambia
Neonatal tetanus protection	99	90 (85-94)	60	99	Central African Republic, Nigeria	Dominican Republic, Eritrea, Guyana, Honduras, Sri Lanka, Maldives, Sao Tome and Principe
Birth						
Skilled attendant at delivery	109	92 (74-99)	20	100	Chad	Turkmenistan, Uzbekistan
Postnatal						
Postnatal visit for mothers	68	71 (50-90)	1	100	Colombia	Turkmenistan
Postnatal visit for babies	61	64 (32-91)	4	100	Chad	Turkmenistan
Early initiation of breastfeeding	68	54 (41-67)	20	90	Pakistan	Sri Lanka
Infancy						
Exclusive breastfeeding (<6 months)	68	46 (35-58)	0	87	Chad	Rwanda
Continued breastfeeding (year 1)	67	80 (60-90)	25	98	Serbia	Nepal
DTP3 immunisation	137	90 (81-96)	25	99	Equatorial Guinea	Albania, China, Cuba, Fiji, Iran, Sri Lanka, Morocco, Maldives, Mongolia, Malaysia, Turkmenistan
Measles immunisation (first dose)	137	89 (78-96)	30	99	Equatorial Guinea	China, Cape Verde, Cuba, Eritrea, Iran, Kazakhstan, Sri Lanka, Morocco, Maldives, Mongolia, Mauritius, Nicaragua, Nauru, Rwanda, Turkmenistan, Tanzania, Saint Vincent and the Grenadines
Rotavirus immunisation	73	85 (74-92)	2	99	Philippines	Fiji, Morocco
Childhood						
Vitamin A supplementation (two doses)	66	62 (26-90)	0	99	Burkina Faso, Central African Republic, Gabon, Mauritania	Benin, Bangladesh, Morocco, Uzbekistan, Vietnam, Zambia
Care seeking for pneumonia symptoms	66	63 (49-77)	24	93	Nigeria	Cuba
Diarrhoea: oral rehydration treatment	64	44 (34-55)	16	84	Cameroon	Eswatini
Environment						
Population using basic drinking water services	138	91 (69-97)	39	100	Chad	Romania
Population using basic sanitation services	135	76 (39-91)	7	100	Ethiopia	Libya, Uzbekistan

* Includes Panama, which was reclassified as a high income country in 2018. Data from WHO and Unicef on immunisation rates, July 2019; WHO and Unicef Joint Monitoring Programme for Water Supply and Sanitation, June 2019; Joint United Nations Programme on HIV/AIDS, July 2019; and Unicef (www.data.unicef.org ) and WHO global databases (www.who.int/gho/database/en/ ), May 2019 (based on demographic and health surveys, multiple indicator cluster surveys, and other national surveys)

## Have we progressed, stumbled, or flatlined?

**Fig 2:**
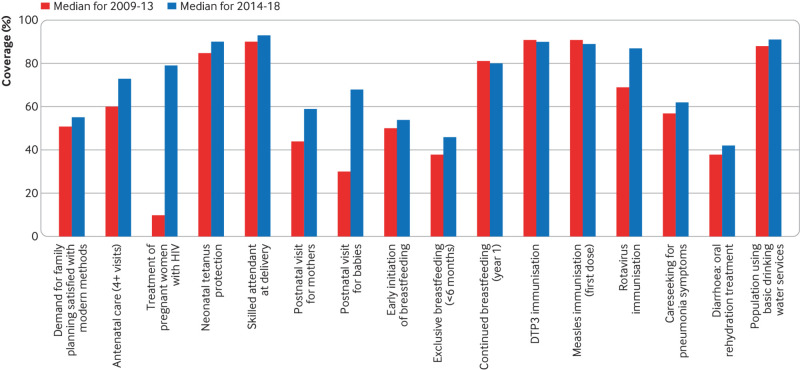
Median national coverage of selected interventions for women’s and child health for 2009-13 and 2014-18, among countries with data for both periods (DTP3=diphtheria-tetanus-pertussis)

We evaluated change in coverage over the past 10 years, focusing on countries with data available for 2009-13 and 2014-18 (table 2, fig 2). We included only countries with at least one estimate for each time interval. Thirteen out of the 16 interventions examined show positive change over the past 10 years, especially treatment of pregnant women with HIV, which has benefited from substantial global investment. Three interventions showed no improvement or a decline: diphtheria-tetanus-pertussis (DTP3) vaccination, measles vaccination, and number of children aged 12 to 15 months who are still breastfed. Because rotavirus vaccine is relatively new, immunisation increased substantially between the two periods as countries implemented the policy. However, universal coverage has not been achieved, and coverage levels are around the same as for DTP3 and measles.

**Table 2:** Changes in median national coverage of selected interventions in low and middle income countries with available data for 2009-13 and 2014-18, ordered by proportion of the gap closed

Stage	Indicator	No of countries	2009-13 median	2014-18 median	Change (% points)	% of gap closed
Pregnancy	Treatment of pregnant women with HIV	93	10	79	69	77
Infancy	Rotavirus immunisation	34	69	87	18	58
Postnatal	Postnatal visit for babies	31	30	68	38	54
Pregnancy	Neonatal tetanus protection	99	85	90	5	33
Pregnancy	≥4 antenatal contacts	63	60	73	13	32
Birth	Skilled attendant delivery	95	90	93	3	30
Postnatal	Postnatal visit for mothers	38	44	59	15	27
Environment	Population using basic drinking water services	138	88	91	3	25
Infancy	Exclusive breastfeeding (<6 months)	52	38	46	8	13
Childhood	Care seeking for pneumonia symptoms	54	57	62	5	12
Pre-pregnancy	Demand for family planning satisfied with modern methods	62	51	55	4	8
Postnatal	Early initiation of breastfeeding	53	50	54	4	8
Childhood	Oral replacement therapy for diarrhoea	56	38	42	4	6
Infancy	Continued breastfeeding to 1 year	51	81	80	−1	−5
Infancy	DTP3 immunisation	137	91	90	−1	−11
Infancy	Measles immunisation (first dose)	137	91	89	−2	−22

Most recent data point for the 2014-18 period and earliest data point for the 2009-2013 period. For countries that had more than one survey during 2009-13, we selected the survey that was closest to the midpoint of 2011. If surveys were equidistant in time to the midpoint, we chose the earlier survey. See web supplement for dates and information on how the percentage coverage gap closed was calculated.

The data suggest that immunisation coverage is flatlining. Many countries experience challenges over vaccines costs, vaccine supply chains, and difficulties reaching the most disadvantaged children in remote or rural areas, or in slums. In some countries parental willingness to vaccinate is also a problem. Although considerable attention has been devoted to understanding vaccine hesitancy in high income settings, most unvaccinated children live in areas without reliable access to them.^9^

Further scrutiny is needed to understand the factors driving positive trends in low and middle income countries as well as those that are inhibiting progress so that appropriate action can be taken. Interventions with high national coverage can start to fall behind as focus shifts elsewhere. Gains need to be sustained through close partnerships between the global community, governments, and local civil society groups.

## Best and worst performing countries

Indices including standardised indicators are useful for comparing overall country performance towards specific development goals. Such comparisons can help to identify which countries are progressing and which are falling behind and should trigger additional national and sub-national analyses to understand the factors driving success and failure.

We used the composite coverage index (CCI)^10^ to rank countries with available data on every indicator included in the index during 2010-18 (45 countries lack available data, most of which are middle income countries in Europe, North America, Oceania, and Latin America and the Caribbean). The CCI is an established summary measure for intervention coverage that correlates with key indicators of health status such as child mortality and stunting. It is calculated from data provided by UN member states, so the quality of the measure is only as strong as the underlying data.

The index has four components (reproductive health, maternal health, child immunisation, and treatment of childhood illness), is limited to interventions delivered through healthcare systems, and has eight coverage indicators: access to modern family planning methods, four or more antenatal care visits, skilled attendant at birth, three doses of DTP vaccine, BCG vaccination, first dose of measles vaccine, oral rehydration salts for diarrhoea, and care seeking for acute respiratory infections (see web supplement for details on how the index is calculated).

**Fig 3:**
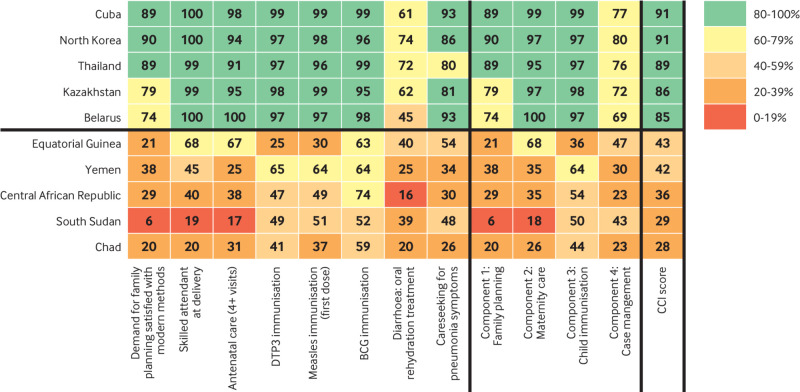
Heatmap of the composite coverage index (CCI) for the five best and worst performing low and middle income countries with available data, 2010-18, showing values for constituent indicators and four components of the index

Figure 3 shows a heatmap of the CCI for the five best and five worst performing countries (a heat map for all low and middle income countries is given in the web supplement). Indicators for the best performers are mostly green, but all have slightly lower coverage for treating childhood illness. The indicators for the lowest performers, in contrast, are mostly red and orange, with some performing better for immunisations. A comprehensive approach is clearly needed for these countries to increase access to care for women and children.

The best performers are spread across different world regions, but apart from Yemen the lowest performers are all in Africa, suggesting that greater attention needs to be directed there. Comparing these countries’ performance on the CCI to mortality statistics provides support for the role of this evaluation framework and the role of high intervention coverage in improving survival. The highest performers have all achieved considerable reductions in maternal and child mortality over the past 10 years.^11^ ^12^ The poorest performers, in contrast, have made less progress in improving maternal and child survival; all but Equatorial Guinea have levels of stunting of at least 30%, and four of the five (Central African Republic, Chad, South Sudan, and Yemen) are affected by conflict or are fragile states.^13^ ^14^

## Where do we go from here?

Global monitoring involves regular tracking of sentinel indicators to assess progress towards fixed goals and targets. But global monitoring is also an iterative process, staying on track with changes in the evidence on effective interventions, implementation strategies, demographic and epidemiological trends, and country priorities. Thus, indicators monitored through global initiatives need to be regularly reviewed and updated.

The SDG and Every Woman Every Child strategy frameworks use a life course approach to development, which has been reflected in strategic plans, programming, and research agendas. The large reduction in child mortality^11^ has enabled a shift from a focus on reducing deaths from infectious causes and malnutrition in young children to tackling health and development needs throughout the first two decades of life.

Improvements in maternal mortality from direct causes means that indirect causes such as reduced fertility and women having their first pregnancies at older ages are becoming more important.^12^ The focus of action is therefore shifting from improving access to intrapartum and emergency obstetric care to include women’s experiences of care, the quality of available services, short and long term forms of maternal morbidity and disability, and women’s empowerment, social status, and rights.

Available evidence also shows huge variations in countries’ progress on the epidemiological, demographic, and nutritional transitions and hence “one size fits all” approaches to development will not work. Poverty reduction is not a straight trajectory, and success can be precarious. Conflict, global recessions, in-country economic crises, environmental disasters, and epidemics can all cause massive destruction, particularly if resilience and safety nets are not built into health and other systems. Several countries were downgraded, for example, in the 2019 World Bank income classifications, with implications for the resources they have for social protection, health, and other welfare programmes.^6^ Hence, the international community and country governments must remain vigilant and rapidly respond to deteriorating situations as part of the pledge to achieve sustainable development.

The challenge in selecting core indicators for global monitoring is to keep the number small so that reporting burdens on countries are manageable yet comprehensive enough to be relevant and spur action. We cannot lose sight of the fact that the leading killers of young children stubbornly remain pneumonia, diarrhoea, and malaria (in endemic areas), often underpinned by undernutrition. But the indicator set needs to encompass children aged 5-9 years, along with other emerging priorities relating to chronic diseases, disabilities, injuries, violence, and child development.

Adolescent health has garnered considerable attention in recent years.^15^ However, only a few adolescent specific indicators have been included in global monitoring frameworks, mainly around reproductive and maternal health, and most are not included as part of routine data collection activities, which hampers accountability.^16^

In response to evidence documenting an increasing proportion of child deaths in the neonatal period, numerous initiatives have aimed to improve monitoring around maternal and newborn health, including health systems, community, and broader social, political, and environmental determinants, and the design and implementation of effective programmes.^17^ ^18^ This has also prompted greater focus on the quality of care provided before, during, and immediately after delivery and motivated the development of guidance on assessing health facility readiness to provide high quality care and health workers’ performance.^19^

The importance of a life course approach has become internationally recognised, as has the salience of viewing women’s, children’s, and adolescents’ health as interlinked and having intergenerational effects. However, data collection efforts are a few steps behind, with data gaps and measurement challenges persisting. Even for those indicators that cover well established interventions, many countries are missing data from the past five years (tables 1 and 2). Most of the 139 countries have modelled data for established interventions such as immunisations, water and sanitation, and HIV. The number of countries with household survey based data range from 61 (for postnatal care for babies) to 109 (for skilled attendant at birth). This wide range in data availability is due to a combination of factors—some of the interventions are new or have never been included in country programmes or data collection processes (eg, rotavirus vaccine, postnatal care for babies) but many of the 139 countries have not conducted a household survey in the past five years. As countries develop and move from low income to middle income status, they often decide to carry out their own national household surveys, which do not always follow standardised methods (such as the international demographic and health surveys). These surveys generate non-comparable data, limiting inclusion in global databases. Conflict or other circumstances can also preclude nationally representative household surveys, and some countries have not provided the resources to carry out surveys.

Global stewardship is now needed on several fronts:

- To increase data collection efforts and to generate more comparable data
- To increase investments in countries’ health information systems and promote better use of existing data
- To coordinate existing initiatives on updating core lists of indicators to monitor progress towards the 2030 goals
- To refine and move forward the measurement agenda, and
- To push for accountability so that every woman, child, and adolescent gets the care they deserve.

Promising efforts are under way to close data gaps for women, children, and adolescents.^16^ ^20^ WHO and Unicef have engaged experts to reach consensus on how to define and measure effective coverage in order to better capture the potential effect health services can have on health and nutrition outcomes.^21^ Many of the indicators included in global monitoring frameworks and in the continuum of care chart (fig 1) capture information on service contacts and do not provide information on quality of care, which is desperately needed.

A consultative process for revising the continuum of care chart should incorporate all these changes on the global landscape, technical advancements in measurement, and country realities. Both the Countdown and Every Woman Every Child indicator lists were derived from lengthy consultative processes involving academia, civil society, UN agencies, healthcare professionals, and countries’ ministries of health.^3^ ^5^ A similar process, soon to be instituted, will allow reporting on key indicators of intervention coverage at the midpoint of the SDG period, enabling assessment of whether global progress is on track. This consultative process should be informed by the ongoing work of relevant global accountability and monitoring initiatives.^22^ ^23^ ^24^ The process should examine how best to obtain data for missing or under-represented areas such as children aged 5-9, adolescents aged 10-14, adolescent indicators beyond reproductive and maternal health, environmental indicators beyond water and sanitation, and a wider list of reproductive and sexual health indicators as part of health outcomes for adult women.

The revised chart should still be viewed as embedded within the common evaluation framework, serving as a starting point for further analyses focusing on key drivers of coverage such as equity, policy and legislative frameworks, and contextual and health systems factors. A companion chart on quality of care measures is also being developed.^25^ Unicef and WHO, in partnership with Countdown to 2030, the UN Population Fund (UNFPA), and other UN agencies will lead this consultative process.

## Conclusion

Our analyses show that work is needed to achieve universal coverage of important health interventions and to prevent interventions with high coverage levels, such as immunisations, from backsliding. Indicator lists and reporting processes must evolve so that they remain relevant to guide national action and programmatic responses. We have suggested a consultative process for revising Countdown’s continuum of care chart to bring it up to date with new thinking on life course approaches and to ensure a manageable set of indicators for reporting on the survive, thrive, and transform dimensions of the Every Woman Every Child strategy. We believe greater investment in data collection is needed so that all countries can report on a core set of indicators, enabling meaningful assessments of global progress.

Finally, further disaggregation of intervention coverage by key markers of equity and the application of newer techniques such as geospatial analyses are essential to improve our understanding of who is being left behind. This information is the starting point for designing strategies to reach all women and children, and for holding all to account for successful implementation.

**Key messages**

- Despite substantial progress in reducing maternal, newborn, and child mortality worldwide, inequities persist
- Countries in sub-Saharan Africa are lagging most behind.
- Coverage of interventions is higher for those that are well resourced, can take place at planned times (such as preventive services), and do not depend on a functioning healthcare system
- The indicators for monitoring progress need to be revised to include proved interventions for older children, adolescents, and adult women
- Further disaggregation of intervention coverage by equity measures is important to better understand who is being left behind

## Web extra

Extra material supplied by authors

Web supplement: Additional information on indicator lists for the Every Woman Every Child global strategy and Countdown to 2030, the evaluation framework underlying the analyses, the methods used to prepare tables and figures, plus supplemental figures.

## Notes

Contributors and sources: JR, TD, DC, DJ, ABM, RG, KS conceived the outline of this paper and drafted the first version, LP and AC conducted analysis of data to create the CoC and CCI. DC, DJ, ABM, RG, KS, ACM, LS, JPM, and AB reviewed and revised drafts of the manuscript, and JR and TD created the final version. The data used for the analyses come from publicly available Unicef and WHO global databases, much of which comes from the Demographic and Health Survey (https://dhsprogram.com) and Multiple Indicator Cluster Survey (https://mics.unicef.org/) programmes.

## Notes

Data availability: Data are available from the authors on reasonable request, including the underlying data files used to generate the figures and the associated code.

Provenance and peer review: Commissioned; externally peer reviewed.
