# Supplementary material for: Assessing coverage of interventions for reproductive, maternal, newborn, child, and adolescent health and nutrition
Source: BMJ. 2020 Jan 26;368:l6915. doi: 10.1136/bmj.l6915 (PMC7461908; doi:10.1136/bmj.l6915)
Supplement: Supplementary file 1 — Web supplement: Additional information on indicator lists for the Every Woman Every Child global strategy and Countdown to 2030, the evaluation framework underlying the analyses, the methods used to prepare tables and figures, plus supplemental figures. [file reqj51682.ww1.pdf]

Web supplement

The annex is organized into two parts. The first includes more information on the indicator lists for the Global Strategy and Countdown to 2030, the evaluation framework underlying the analyses, and the methodology used to prepare tables and figures in the paper. The second part includes supplemental figures.

1. ***Indicator lists and evaluation frameworks***
   a. Countdown to 2030

The Countdown initiative undertakes a rigorous technical review process approximately every two years. The purpose of the technical review process is to ensure that the Countdown indicator list is updated to reflect any changes in the evidence base on effective interventions, improvements in data collection and measurement, and country and global priorities. The last technical review process was completed in 2017 and resulted in the country and indicator lists used for the 2017 country profiles and the analyses in the Countdown 2017 report. A comprehensive description of the steps involved and results of the technical review process are available on the Countdown website: http://countdown2030.org/country-and-regional-networks/country-profiles/countdown-to-2030-data-sources-and-methods-december-2017-country-profiles.

The Countdown analyses are based on a modification of the common evaluation framework. A graphical depiction of the framework is available on page 20 of the technical review process document.

Our article calls for a consultative technical review process to revise the Countdown and Global Strategy indicator lists, country list, and evaluation framework in view of the shift in orientation towards a life course perspective

   b. Global Strategy for Women's, Children's and Adolescents' Health

The monitoring framework for the Global Strategy was developed as a one-time process for selecting the set of indicators to monitor the Strategy's thrive, strive and transform dimensions. The H6 agencies collaboratively report on the indicators in the framework through the Global Strategy data portal available on the WHO's global health observatory: https://apps.who.int/gho/data/node.gswcah

Both the Countdown to 2030 and Global Strategy monitoring frameworks are derived from the common results framework which is described in detail in WHO's Monitoring Framework for Health System Strength.

https://www.who.int/healthinfo/HSS_MandE_framework_Nov_2009.pdf

2. ***Methods***

a. Explanation of the sources and the year of the data for each indicator included in Figure 1, Figure 2, and Table 1.

- Immunizations. These data are from the WHO and UNICEF joint estimates, last update in July, 2019. The year of the data is 2018.
- Population using basic drinking water services and basic sanitation services. These data are from the WHO and UNICEF Joint Monitoring Programme for Water Supply, Sanitation and Hygiene, last update June 2019. The year of the data is 2017.
- Pregnant women living with HIV receiving antiretroviral treatment for PMTCT. These data are from the Global AIDS Monitoring and UNAIDS 2019 estimates, last update July 2019. The year of the data is 2018.
- All other indicators come from the UNICEF and WHO global databases, last updated in May 2019. These data are based on Demographic and Health Surveys, Multiple Indicator Cluster Surveys and other national surveys. Only countries with available data from the time period 2014-2018 are included.

b. Explanation of how the coverage gap closed was calculated

The coverage gap is defined as 100% (complete/full coverage) minus the coverage level in the earlier time period. The coverage gap closed (CGC) for each intervention is a proportion, calculated from the following formula, where C2 is defined as the median global coverage in the most recent period and C1 defined as the median global coverage in the first or earlier period: %CGC = 100 * $(\frac{C2-C1}{100-C1})$

c. Alternate approach to assessing changes over time

- We opted to use median rather than the mean as the measure of central tendency to be consistent with previous Countdown to 2030 and Countdown to 2015 analyses. The findings yielded similar results about intervention progress, so the choice of measure, mean or median, does not affect the main messages.

- Rather than using the coverage gap closed measure to assess progress over time and to compare which interventions are progressing faster than others, an approach that is based on calculating the average annual percentage point change for each indicator can be undertaken.

The formula for this is:

Global annual percentage point change = median of all percentage point change, calculated for each country (see below for calculation)

Country annual percentage point change = $\frac{Percentage\ point\ change\ between\ two\ time\ periods\ (for\ specific\ country)}{Number\ of\ years\ between\ two\ time\ periods\ (for\ specific\ country)}$

When we used this approach, the findings result in the same messages as the coverage gap closed approach. Namely, the two approaches show the same interventions as having the most progress, and the same interventions that are lagging the most behind.

In the first step to compare the two methodological approaches, we first examined if there were systematic differences in the average number of years between the first and second surveys for the indicators. We found that for the indicators for which modeled estimates are available, the average number of years was 7. This was slightly lower for the indicators based on survey data (around 5). (slides 2-4)

The second step was to determine if the measure of central tendency used made a difference in determining the annual change in coverage by indicator. The results found no difference (slides 5 and 6).

The third step involved comparing the findings of the average annual percentage point change for each indicator compared to the bar chart showing the changes in the median coverage in the two time periods. (slides 8-10). Although the numbers differ, the main messages are the same – the two methods identify the same faster and slower progressing interventions. We have opted to include the bar chart showing the two medians in the two time intervals as we think this is more visually understandable to most audiences.

d. Computation of the composite coverage index (CCI)

The Composite coverage index includes four components and eight indicators:

i) reproductive health: measured through demand for family planning satisfied with modern methods among women of reproductive age (DFPSm)
ii) maternal health: measured as the arithmetic average of the two indicators of four or more antenatal care visits (ANC4) and skilled birth attendant (SBA)
iii) child immunization: measured as a weighted average of three doses of diptheria, tetanus and pertussis (DTP3 – multiplied by 2 in the formula), Bacillus Calmette-Guerin (BCG), and first dose of measles (MCV1)
iv) child illness treatment: measured as the arithmetic average of ORS for diarrhea and careseeking for acute respiratory infection from a formal provider (CAREP)

The formula used to compute the CCI is:

$$CCI = \frac{1}{4}\left(DFPSm + \frac{ANC4 + \text{SBA}}{2} + \frac{\text{BCG} + 2 \times DPT3 + MCV1}{4} + \frac{ORS + CAREP}{2}\right)$$

For more information on the CCI, see: Wehrmeister FC, Restrepo-Mendez MC, Franca GV, et al. Summary indices for monitoring universal coverage in maternal and child health care. *Bull World Health Organ* 2016;94(12):903-12. doi: 10.2471/BLT.16.173138 [published Online First: 2016/12/21]

3. ***Supplemental Figures:***

The first two figures present variations of the continuum of care chart (figure 1 in the main text) to illustrate how intervention coverage varies by income level of the country, and by SDG region. The heat map included in the paper itself presents data for the 5 best and 5 worst performers on the CCI. The third figure in the annex presents the CCI information for all of the 94 countries that had comparable data for each indicator included in the CCI, time period of 2010-2018.

ANNEX FIGURE 1. Median national coverage of select interventions along the continuum of care (CoC), most recent survey for each country with data available, 2014 and later (%); bars represent global median, median among low income countries in black diamonds, median among lower-middle income countries in grey diamond, and median among upper-middle income countries in white diamond

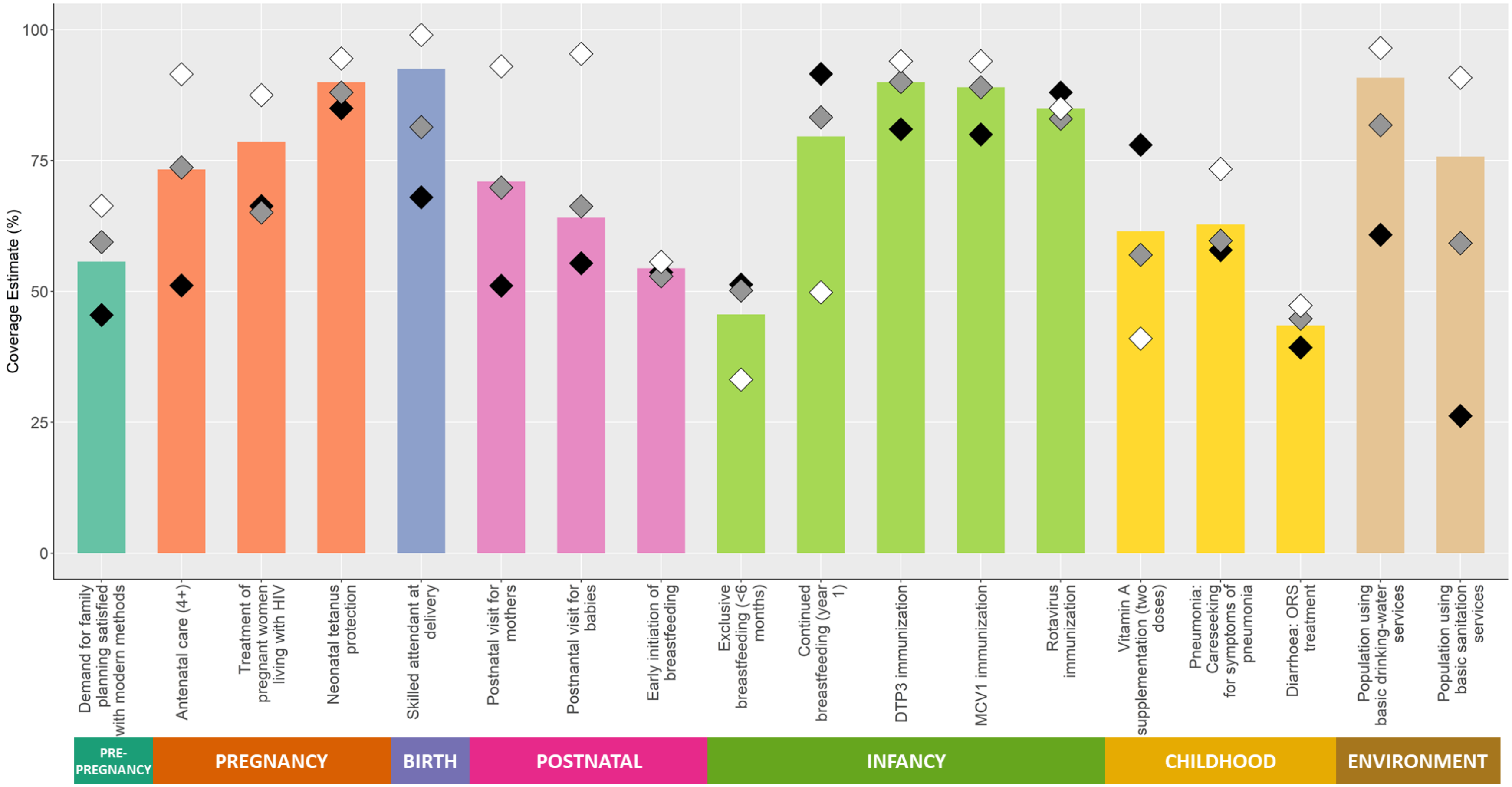

ANNEX FIGURE 2. Median national coverage of select interventions along the continuum of care (CoC), most recent survey for each country with data available, 2014 and later (%); bars represent global median, regional medians represented by diamonds

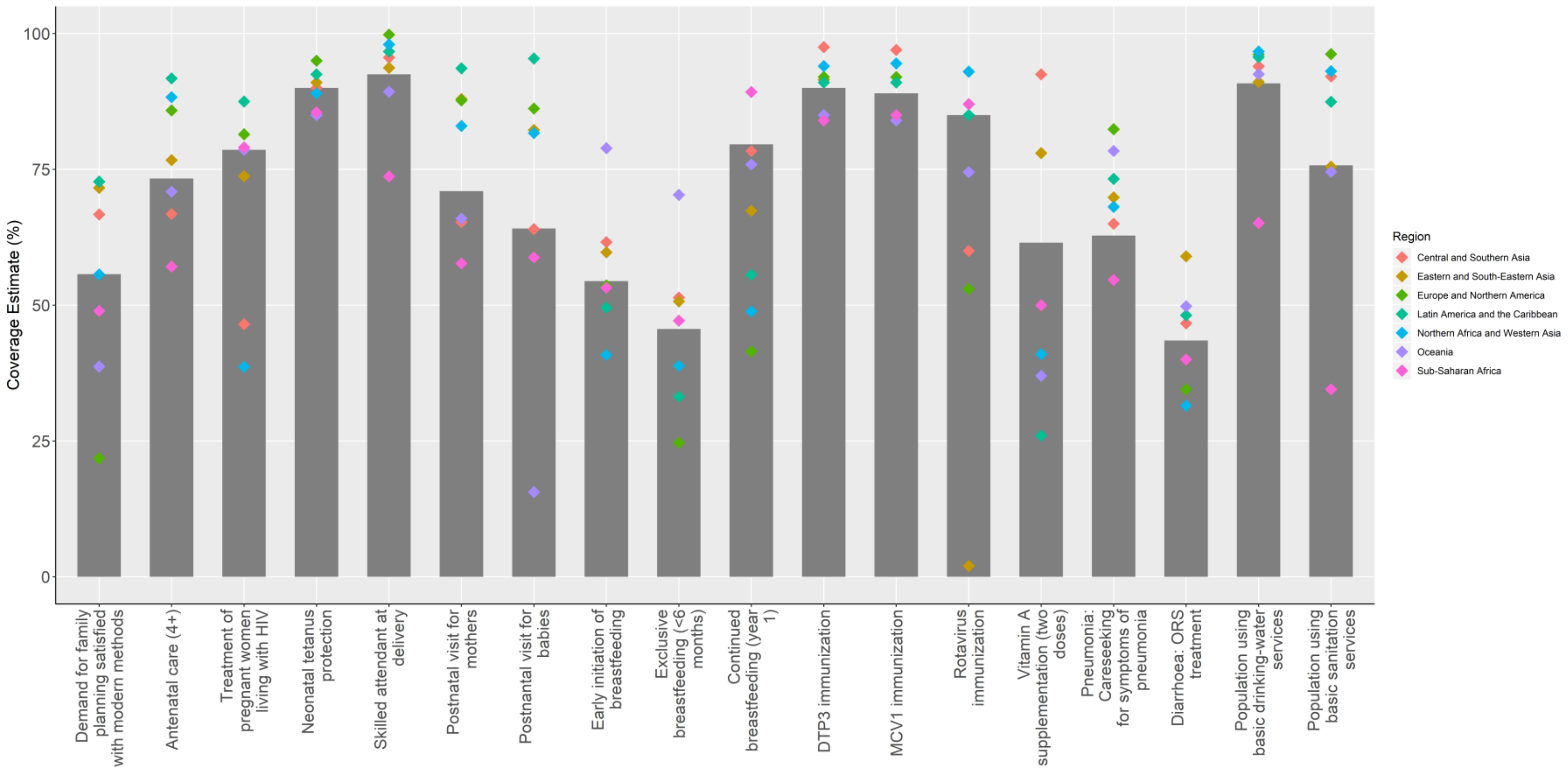

ANNEX FIGURE 3. A heatmap of the Composite Coverage Index, its four components, and the constituent indicators of the components, among 94 countries with complete data available, sorted by high to low on the CCI*

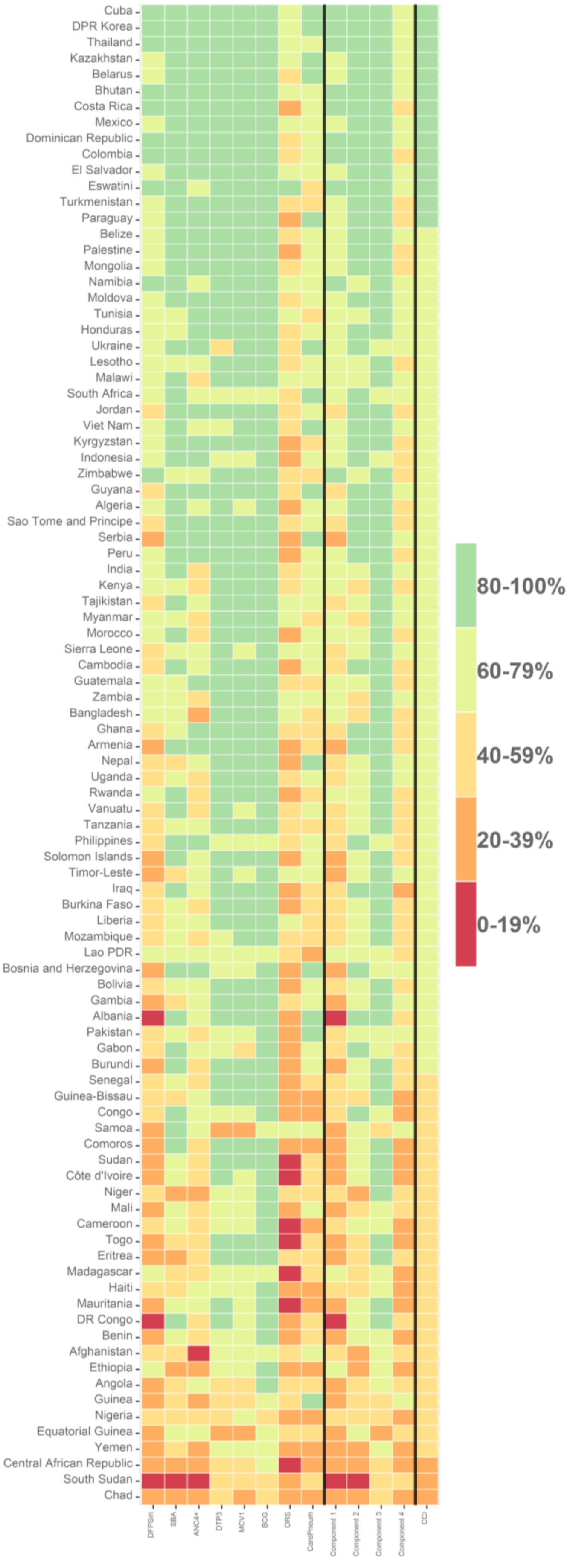

Country	DFPSm	SBA	ANC4+	DTP3	MCV1	BCG	ORS	CarePneum	Component 1	Component 2	Component 3	Component 4	CCI
Cuba	89	100	98	99	99	99	61	93	89	99	99	77	91
DPR Korea	90	100	94	97	98	96	74	86	90	97	97	80	91
Thailand	89	99	91	97	96	99	72	80	89	95	97	76	89
Kazakhstan	79	99	95	98	99	95	62	81	79	97	98	72	86
Belarus	74	100	100	97	97	98	45	93	74	100	97	69	85
Bhutan	85	96	85	97	97	99	61	74	85	91	96	68	85
Costa Rica	89	99	90	94	94	92	40	77	89	94	94	59	84
Mexico	80	98	94	88	97	96	61	73	80	96	92	67	84
Dominican Republic	82	100	93	94	95	99	48	73	82	96	96	60	84
Colombia	87	99	90	92	95	89	54	64	87	95	92	59	83
El Salvador	80	100	90	81	81	81	70	80	80	95	81	75	83
Eswatini	83	88	76	90	89	98	84	60	83	82	92	72	82
Turkmenistan	76	100	96	99	99	98	47	59	76	98	99	53	81
Paraguay	79	97	94	88	93	91	28	89	79	95	90	59	81
Belize	66	92	93	96	97	99	55	67	66	92	97	61	79
Palestine	65	99	96	99	99	99	32	76	65	97	99	54	79
Mongolia	65	98	90	99	99	99	42	70	65	94	99	56	79
Namibia	80	88	62	89	82	94	72	68	80	75	86	70	79
Moldova	60	100	95	93	93	96	42	79	60	98	94	60	78
Tunisia	73	74	85	97	96	92	65	60	73	79	96	62	78
Honduras	76	74	89	90	89	94	60	64	76	81	91	62	78
Ukraine	68	100	87	50	91	90	59	92	68	94	70	76	77
Lesotho	79	78	74	93	90	98	53	63	79	76	94	58	77
Malawi	74	90	51	92	87	92	65	78	74	70	91	71	76
South Africa	78	97	76	74	70	70	51	88	78	86	72	70	76
Jordan	57	100	92	96	92	94	44	72	57	96	94	58	76
Viet Nam	70	94	74	75	97	95	51	81	70	84	86	66	76
Kyrgyzstan	66	98	95	94	96	97	33	60	66	96	95	47	76
Indonesia	78	94	84	79	75	81	39	75	78	89	78	57	75
Zimbabwe	85	78	76	89	88	95	40	51	85	77	90	46	74
Guyana	52	86	87	95	98	99	42	84	52	86	97	63	74
Algeria	77	97	67	91	80	99	25	66	77	82	90	46	74
Sao Tome and Principe	52	92	84	95	95	96	49	69	52	88	95	59	74
Serbia	39	98	94	96	92	98	36	90	39	96	96	63	73
Peru	67	93	96	84	85	81	32	62	67	94	84	47	73
India	67	81	51	89	90	92	51	78	67	66	90	64	72
Kenya	76	62	58	92	89	95	54	66	76	60	92	60	72
Tajikistan	45	95	64	96	98	99	62	68	45	80	97	65	72
Myanmar	75	60	59	91	93	90	62	58	75	59	91	60	71
Morocco	69	87	55	99	99	99	22	70	69	71	99	46	71
Sierra Leone	45	69	78	90	80	90	78	74	45	73	86	76	70
Cambodia	56	89	76	92	84	93	35	69	56	82	90	52	70
Guatemala	66	69	86	86	87	88	49	52	66	78	87	50	70
Zambia	62	63	56	90	94	91	64	70	62	59	91	67	70
Bangladesh	73	68	31	98	97	99	77	42	73	50	98	60	70
Ghana	46	78	89	97	92	98	49	53	46	84	96	51	69
Armenia	37	100	96	92	95	99	37	57	37	98	94	47	69
Nepal	56	58	69	91	91	96	37	85	56	64	92	61	68
Uganda	54	74	60	93	86	88	44	80	54	67	90	62	68
Rwanda	63	91	44	97	99	97	28	54	63	67	98	41	67
Vanuatu	51	89	52	85	75	94	48	72	51	71	85	60	66
Tanzania	54	64	62	98	99	99	45	55	54	63	98	50	66
Philippines	52	84	86	65	67	75	45	67	52	85	68	56	66
Solomon Islands	38	86	69	85	93	83	37	79	38	78	86	58	65
Timor-Leste	37	57	77	83	77	95	70	71	37	67	84	70	65
Iraq	55	96	68	84	83	95	25	44	55	82	86	35	64
Burkina Faso	56	80	47	91	88	98	40	52	56	64	92	46	64
Liberia	41	61	78	84	91	92	60	51	41	70	88	56	64
Mozambique	56	73	52	80	85	95	46	56	56	62	85	51	64
Lao PDR	72	64	62	68	69	79	56	40	72	63	71	48	63
Bosnia and Herzegovina	22	100	84	73	68	95	36	87	22	92	77	62	63
Bolivia	50	71	75	83	89	90	22	62	50	73	86	42	63
Gambia	27	57	78	93	91	94	59	68	27	67	93	64	63
Albania	5	100	78	99	94	99	34	82	5	89	98	58	62
Pakistan	48	69	51	75	76	86	37	84	48	60	78	61	62
Gabon	44	89	78	70	59	87	26	68	44	83	72	47	61
Burundi	38	85	49	90	88	91	36	62	38	67	90	49	61
Senegal	51	68	57	81	82	83	28	60	51	63	82	44	60
Guinea-Bissau	56	45	65	88	86	91	35	34	56	55	88	35	58
Congo	43	91	79	75	75	81	27	28	43	85	76	28	58
Samoa	39	82	73	34	31	62	63	78	39	78	40	70	57
Comoros	29	82	49	91	90	94	38	38	29	66	92	38	56
Sudan	30	78	51	93	88	88	20	48	30	64	90	34	55
Côte d'Ivoire	39	74	51	82	71	98	16	44	39	62	83	30	54
Niger	46	40	38	79	77	87	41	59	46	39	80	50	54
Mali	35	67	43	71	70	83	21	71	35	55	74	46	53
Cameroon	47	65	59	79	71	88	16	28	47	62	79	22	52
Togo	37	45	57	88	85	83	19	48	37	51	86	34	52
Eritrea	21	34	57	95	99	97	43	45	21	46	96	44	52
Madagascar	60	44	51	75	62	70	15	40	60	48	70	28	52
Haiti	43	42	67	64	69	83	39	37	43	54	70	38	51
Mauritania	30	69	63	81	78	90	19	34	30	66	82	26	51
DR Congo	19	80	48	81	80	83	39	42	19	64	81	40	51
Benin	26	78	52	76	71	89	22	46	26	65	78	34	51
Afghanistan	42	59	18	66	64	78	46	62	42	38	68	54	51
Ethiopia	62	28	32	72	61	85	30	31	62	30	72	30	49
Angola	30	47	61	59	50	86	43	49	30	54	64	46	48
Guinea	22	55	35	45	48	72	55	83	22	45	52	69	47
Nigeria	43	43	49	57	65	53	37	24	43	46	58	30	44
Equatorial Guinea	21	68	67	25	30	63	40	54	21	68	36	47	43
Yemen	38	45	25	65	64	64	25	34	38	35	64	30	42
Central African Republic	29	40	38	47	49	74	16	30	29	39	54	23	36
South Sudan	6	19	17	49	51	52	39	48	6	18	50	43	29
Chad	20	20	31	41	37	59	20	26	20	26	44	23	28

80-100%
60-79%
40-59%
20-39%
0-19%

***Annex Figure 3. Con't. Acronyms defined**

The heatmap includes information on each of the indicators included in the CCI components. Here is the full indicator name represented by acronyms in the chart:

1. DFPSm: Demand for family planning satisfied with modern methods
2. SBA: Skilled attendant at delivery
3. ANC4+: Antenatal care (4 or more visits)
4. DTP3: Three doses of diptheria, tetanus and pertussis vaccines
5. MCV1: First dose of measles vaccine
6. BCG: Bacillus Calmette-Guerin vaccine
7. ORS : Oral rehydration salts
8. CarePneum : Care seeking for pneumonia
